# 임신 중 성에 대한 태도가 임신 부부의 성기능에 미치는 영향

**DOI:** 10.4069/kjwhn.2020.06.18.1

**Published:** 2020-06-24

**Authors:** Moon Jeong Kim

**Affiliations:** Department of Nursing, Pukyong National University, Busan, Korea; 부경대학교 간호학과

**Keywords:** Attitude, Pregnancy, Sexual behavior, 태도, 임신, 성행위

## Abstract

**Purpose:**

This study was conducted to identify the influences of the attitudes of pregnant women and their husbands towards sex during pregnancy on sexual function. Additional purpose was to compare the frequency of sexual dysfunction according to gender and gestational trimester and to describe the changes in sexual behavior according to the gestational trimester.

**Methods:**

In this study, 231 pregnant couples completed self-report questionnaires during their visits to women’s hospitals or community health centers. The questionnaires assessed general characteristics, maternal/paternal sexual attitudes towards sex during pregnancy (Maternal Sex during Pregnancy Scale, MSP/Paternal Sex during Pregnancy Scale, PSP), and sexual function (using the Female Sexual Function Index and International Index of Erectile Function, respectively). Multiple regression analysis was done to test the research model using SPSS version 23.0.

**Results:**

In this study, 74.9% of pregnant women and 38.5% of their husbands reported sexual dysfunction. Sexual dysfunction was prevalent in pregnant women in the first trimester and prevalent in husbands in the third trimester. MSP (β=.44, *p*<.001) in pregnant women and PSP (β=.39, *p*<.001) and being in the first trimester (β=.17, *p*=.012) in husbands influenced sexual function during pregnancy. In the first trimester, the scores for attitudes towards sex during pregnancy were the lowest in pregnant women, while they were the highest in their husbands.

**Conclusion:**

Positive attitudes about sex during pregnancy are important for sexual function in pregnant couples. Because the difference in attitudes towards sex during pregnancy between pregnant women and their husbands was greatest in the first trimester, sexual health interventions need to be provided in early pregnancy.

## Introduction

### 연구 필요성

임신 중 생리적, 심리적 변화가 여성의 성기능이나 성 만족에 영향을 미친다는 점은 널리 받아들여지고 있는 사실이다. 임신 여성의 성기능은 비임신 여성에 비해 낮으며, 성기능 부전의 비율도 비임신 여성이 67.6%임에 비해 임신 여성은 79.0–91.1%로 높게 보고되고 있다[1,2]. 성기능 부전은 1, 2, 3삼분기에 각각 23.4%, 30.5%, 46.2%로 임신이 진행됨에 따라 증가하는 것으로 나타났다[1]. 모든 결혼기간에서 성만족은 결혼생활만족도와 관련이 있기 때문에[3] 임신 부부의 성기능은 중요하게 다루어져야 할 문제이다.

착상부터 산후까지 생리적, 심리적, 신체적 영역에서 적지 않은 변화를 고려하면 임신 중 성행위의 변화는 놀랍지 않다. 임신 중 성행위는 문화적 신념 및 금기와 관련이 있고[4], 신체 이미지 인식과 파트너와의 의사소통이 영향을 미치는 것으로 보고된다[5]. 또한 성기능부전의 발생 빈도는 임신 1, 2, 3삼분기에 각각 23.4%, 30.5%, 46.2%로 임신이 경과하면서 증가하는 것으로 나타나[1] 임신기간과 임신 중 성기능의 관련성을 추론해볼 수 있다. 따라서 임신기간에 따라 성행위의 변화, 성기능부전의 발생 빈도를 파악해 볼 필요가 있다.

긍정적인 성 태도를 가진 사람은 성행위의 빈도가 높고, 다양한 성행위를 시도하며, 성기능이 좋은 것으로 보고되었다[6,7]. 임신 부부의 경우에도 마찬가지로 성 태도가 자신과 상대방의 성 만족에 영향을 미치는 것으로 나타났다[8]. 성 태도가 일반적인 성에 대한 호불호나 개방성을 평가하는 개념이라면[9], 임신 중 성에 대한 태도(attitudes towards sex during pregnancy)는 임신 중의 성에 대한 호불호나 개방성을 의미한다고 볼 수 있다. 임신 중 성에 대한 태도는 사회‧문화적 규범, 태아와 임부의 건강과 안전에 대한 우려 등의 영향을 받아 형성되며[10], 2–3삼분기 임부의 성기능에 영향을 미치는 것으로 밝혀졌으나[11], 유산에 대한 우려로 성행위를 기피하는 1삼분기 임부를 대상으로 파악한 연구는 없는 실정이므로 전체 임신 기간에 걸쳐 임신 중 성에 대한 태도를 파악해 볼 필요가 있다.

임신 3기는 임부의 성행위 빈도와 성기능 감소의 중요한 요인으로 보고되었으나[12] 그와 일치하지 않는 결과에 대한 보고들도 있다[2]. 한국 임부의 임신 기간과 성기능의 관계에 대한 경험적 근거는 부족한 실정이다. 한국 임부를 대상으로 한 선행 연구에서 2삼분기와 3삼분기 임부의 성기능의 차이를 보고하였으나[11] 1삼분기 임부에 대한 파악은 이루어지지 않았다. 임신 중 성생활 기피 이유가 주로 유산이나 태아의 건강 손실, 부작용 발생에 대한 두려움인 점을 고려할 때[11], 1삼분기 임부의 성기능과 영향요인에 대한 파악이 필요하다.

성에 대한 인식은 배우자의 욕구, 매력의 느낌, 성교에 대한 두려움에 의해 영향을 받을 수 있으며, 임신 중 성기능의 중요한 부분이다[13,14]. 그러므로 임신으로 인한 아내의 욕구 변화, 아내의 매력에 대한 느낌 변화, 성교가 임신이나 태아에게 미칠 나쁜 영향에 대한 두려움은 남편의 임신 중 성에 대한 태도를 형성하고, 이는 남편의 성기능에 영향을 미칠 수 있다. 남편의 성적 반응은 임신 중에 영향을 받아 아내의 성적 장애를 악화시키고 남편의 성기능 장애를 유발하거나 심화시킬 수 있다. 그러므로 남편을 대상으로 임신 중 성에 대한 태도와 성기능의 정도를 확인하고, 남편의 임신 중 성에 대한 태도가 성기능에 미치는 영향을 확인하는 일은 남편뿐 아니라 부부 모두를 위한 성기능 중재 마련을 위해 필수적이다. 특히 임부와 남편 양측을 대상으로 임신이 경과함에 따라 임신 중 성에 대한 태도와 성기능이 변화하는 양상을 파악한다면 커플의 조화로운 성을 지지하는 성교육 콘텐츠를 마련하는 데에 중요한 시사점을 제공할 수 있을 것이다.

### 연구 목적

본 연구의 목적은 임부와 남편을 대상으로 임신 중 성에 대한 태도가 성기능에 미치는 영향을 규명하는 것이다. 추가적인 목적은 임신기간별로 성행위의 변화를 파악하고, 성별과 임신 기간별로 성기능 부전 정도를 비교하는 것이다. 본 연구의 구체적인 목적은 다음과 같다.

1) 임부와 남편의 일반적 특성에 따른 임신 중 성에 대한 태도와 성기능의 차이를 파악한다.

2) 임부와 남편의 임신 중 성에 대한 태도와 성기능 간의 상관관계를 파악한다.

3) 임부와 남편의 성기능 영향요인을 파악한다.

4) 임부와 남편의 임신 전, 임신기간별 성행위의 변화를 파악한다.

5) 임부와 남편의 임신기간별 성기능부전의 빈도를 비교한다.

## Methods

Ethics statement: This study was approved by the Institutional Review Board (IRB) of Pukyong National University (IRB No. 1041386-20160930-HR-016-03). Informed consent was obtained from the participants.

### 연구 설계

본 연구는 임부와 남편을 대상으로 임신 중 성에 대한 태도가 성기능에 미치는 영향을 규명한 상관관계 연구이다.

### 연구 대상 및 자료 수집 방법

본 연구의 대상자는 울산광역시에 거주하며 임신 합병증이 없는 1–3삼분기 임부와 남편이었다. G*Power 3.1.9를 이용하여 임신 부부의 성기능 영향요인 분석을 위한 다변량 회귀분석과 임신기간별 임신 중 성에 대한 태도와 성기능의 차이 검정을 위한 일원배치분산분석 수행에 필요한 최소 표본의 크기를 구하였다. 유의수준 .05, 검정력 .90, 선행연구[11]에서 중간 효과크기 .15, 예측변인 6개(연령, 학력, 직업 유무, 출산력, 임신기간, 임신 중 성에 대한 태도)의 조건으로 다변량 회귀분석에 필요한 최소 표본 크기는 123명이었다. 그리고 유의수준 .05, 검정력 .90, 중간 효과크기 .25, 세 그룹으로 일원배치분산분석을 실시하는데 필요한 최소 표본 크기는 207명이었다. 탈락율 15%를 고려하여 238부의 자료를 수집하기로 하였다.

자료수집은 임신 부부를 대상으로 2차에 걸쳐 이루어졌다. 1차에서는 142쌍(1삼분기 4쌍, 2삼분기 24쌍, 3삼분기 114쌍), 2차에서는 96쌍(1삼분기 74쌍, 2삼분기 22쌍)의 자료를 수집하였다. 1차 자료수집 시 1삼분기 여성의 참여가 저조하여 대상에 포함하지 않았으며 1삼분기 부부를 포함한 후속연구를 계획하면서 남편의 자료를 분석에서 제외한 후 2–3삼분기 임부의 성기능 영향요인을 분석한 후 보고하였다[11]. 본 연구에서는 선행연구에서 임신 1기 부부가 제외됨으로써 연구결과를 전체 임신 부부에게 적용할 수 없었던 한계점을 극복하고, 임신기간에 따른 성기능부전의 빈도와 성행위의 변화를 함께 파악하고자 하였다.

1차 자료수집은 2017년 8월부터 9월까지, 2차 자료수집은 2017년 11월부터 12월까지 이루어졌다. 1, 2차 자료수집은 모두 울산광역시에 소재한 여성전문병원 2곳과 보건소 2곳에서 이루어졌다. 여성전문병원에서는 간호부장에게, 보건소에서는 보건소장에게 연구의 목적과 조사방법, 연구윤리 준수사항 등을 포함한 연구 전반에 대해 설명한 후 해당 기관에서의 연구 수행에 대한 승인을 얻었다. 여성전문병원의 산부인과 외래와 보건소 출산교실 강의실 벽보에 연구 참여에 대한 안내문을 붙여놓고, 그 아래 연구 설명문, 동의서, 임부용 및 남편용 설문지가 담긴 설문세트를 비치하여 연구대상자가 읽고 자발적 참여가 가능하도록 하였다. 작성이 완료된 설문지는 응답 내용의 기밀 유지를 위해 부부가 개별적으로 밀봉 봉투에 넣어 밀봉하게 하였고, 다음 산전진찰이나 출산교실 참여 시 회수하였다. 1, 2차 자료수집에서 231쌍의 자료가 수집되었고 그 가운데 불성실하게 작성된 7쌍의 설문지를 제거하여 총 231쌍의 자료를 최종 분석에 활용하였다.

### 연구 도구

#### 임신 중 성에 대한 태도

임신 중 성에 대한 태도는 Jawed-Wessel 등[10]의 Maternal Sex during Pregnancy Scale (MSP)과 Paternal Sex during Pregnancy Scale (PSP)을 개발자의 승인을 얻은 후 한국어로 번안한 도구[15]로 측정하였다. MSP와 PSP는 단일 요인이며 공통 항목은 ‘임신 중의 성관계가 어색하다’, ‘성관계를 하면 유산이 될 수 있다’, ‘임신 때문에 성관계에 불안을 느낀다’, ‘임신 때문에 못하는 성교 체위가 있다’, ‘임신 때문에 흥분되는 성생활이 불가능하다’ 등의 5개 항목이다. MSP는 ‘임신 때문에 남편이 내게서 성적 매력을 찾기 어렵다’의 1개 항목이 추가되어 총 6개 항목이고, PSP는 ‘임신 때문에 남편의 성적 매력을 발견하는 것이 어렵다’, ‘임신 때문에 성욕이 잘 생기지 않는다’, ‘임신 동안 성관계보다는 자위가 낫다’ 등 3개 항목이 추가되어 총 8개 항목이다.

MSP와 PSP 모두 6점 Likert 척도로 ‘전혀 아니다’ 1점부터 ‘매우 그렇다’ 6점까지 점수를 부여하였다. 본 연구에서는 해석의 편의를 위해 모든 항목을 역산하였기에 점수가 클수록 임신 중 성에 대한 태도가 긍정적임을 의미한다. 개발 당시 도구의 신뢰도 Cronbach's α 값은 MSP가 .89, PSP는 .91이었다. 본 연구에서는 MSP가 .73, PSP는 .82이었다.

#### 성기능

임부의 성기능을 측정하기 위하여 Lee 등[16]의 Korean version of Female Sexual Function Index-6 (FSFI-6K)를 개발자의 승인을 얻은 후 사용하였다. 이 도구는 지난 4주간 성생활에서 성적 욕구, 성적 흥분, 윤활액 분비, 오르가슴, 성적 만족감, 질 삽입 성교 시 불편감/통증 등 6개 항목에 응답하도록 되어 있다. 점수는 성적 욕구와 성적 만족감에는 1점부터 5점까지, 성적 흥분, 윤활액 분비, 오르가슴과 불편감/통증은 0점부터 5점까지 부여하며 총점은 2점부터 30점까지 가능하고 점수가 클수록 성기능이 좋음을 의미한다. 본 연구에서는 개발 당시 절사점(cutoff score) 21점을 기준으로 성기능 부전을 분류하였다. 도구의 신뢰도 Cronbach’s α값은 개발 당시 .89였고, 본 연구에서는 .85였다.

남편의 성기능을 측정하기 위하여 한국어판 5-item version of the International Index of Erectile Function (IIEF-5)를 사용하였다[17]. 설문지 문항은 발기 기능(erectile function)과 성교 만족도(intercourse satisfaction)에 대한 5개 문항으로 구성되었다. 5점 Likert 척도로 총점은 5–25점까지 가능하고 점수가 클수록 발기능이 좋은 것을 의미하며, 본 연구에서는 한국어판의 절사점 17점을 기준으로 성기능 부전을 분류하였다. 도구의 신뢰도 Cronbach’s α값은 개발 당시 .89였고, 본 연구에서는 .91이었다.

#### 성행위

성행위는 자신의 성을 경험하고 표현하는 방식을 의미하며[18]. 본 연구에서 성행위는 선행 연구[18]를 참고하여 키스, 질 삽입 성교, 전희, 구강 성교, 성적 상상, 자위, 항문 성교를 포함하였다. 임신 중 성생활을 파악하기 위하여 임신 기간별 성행위의 빈도, 성행위를 중단한 이유, 성행위 시 경험한 부작용, 성생활에 관한 정보의 출처 등을 중복 응답하게 하였다.

#### 대상자의 특성

임부와 남편의 일반적 특성으로 연령, 학력, 직업 유무 등 3문항, 산과적 특성은 임부에게만 출산력과 임신기간 등 2문항을 질문하였다.

### 자료 분석

231쌍의 자료를 SPSS v. 23.0 통계 프로그램을 이용하여 분석하였다. 임부와 남편의 일반적 특성에 따른 임신 중 성에 대한 태도와 성기능의 차이는 t-test 또는 ANOVA로 분석하였고, 사후분석은 Scheffé test를 이용하였다. 임부와 남편의 임신 중 성에 대한 태도와 성기능 간의 상관관계는 Pearson’s correlation coefficient로 분석하였고, 임부와 남편의 성기능 영향요인은 multiple linear regression으로 분석하였다. 임부와 남편의 임신 전, 임신기간별 성행위의 변화는 빈도와 백분율로, 임부와 남편 간, 임신기간별 성기능부전 빈도의 비교는 카이검정으로 분석하였다.

## Results

### 임부와 남편의 일반적 특성 및 그에 따른 임신 중 성에 대한 태도와 성기능의 차이

임부의 연령은 평균 32.19±4.09세로 30대가 대부분이었고(71.4%) 학력은 58.9%가 4년제 대학교 졸업 이상이었으며 현재 직업을 가지고 있는 경우는 42.4%였다. 출산력은 대부분 미산부였으며(84.6%) 임신 기간은 평균 24.86±12.35주로 3삼분기가 가장 많았다(49.4%).

임부의 임신 중 성에 대한 태도(MSP)는 6점 만점에 평균 2.91±0.84점이었다. MSP 점수는 학력(t=2.29, *p*=.023), 직업 유무(t=4.92, *p*<.001)와 임신 기간(F=21.54, *p*<.001)에 따른 차이가 유의하였다. 즉 임부의 임신 중 성에 대한 태도는 4년제 대학교 졸업 이상보다 전문대학교 졸업 이하의 학력일 때, 직업이 있을 때보다 없을 때, 1, 2삼분기보다 3삼분기일 때 더 긍정적인 것으로 나타났다.

임부의 성기능(FSFI)은 30점 만점에 평균 12.01±6.79점이었다. FSFI 점수는 학력(t=2.62, *p*=.010)에 따른 차이가 유의하였다. 즉 임부의 성기능은 4년제 대학교 졸업 이상보다 전문대학교 졸업 이하의 학력일 때 더 높았다.

남편의 연령은 평균 35.08±4.30세로 30대가 대부분이었고(76.6%), 학력은 54.4%가 4년제 대학교 졸업 이상이었으며 97.0%가 현재 직업을 가지고 있었다.

남편의 임신 중 성에 대한 태도(PSP)는 6점 만점에 평균 3.47±0.90점이었다. PSP 점수는 학력(t=2.47, *p*=.014)과 임신 기간(F=7.24, *p*=.001)에 따른 차이가 유의하였다. 즉 남편의 임신 중 성에 대한 태도는 4년제 대학교 졸업 이상보다 전문대학교 졸업 이하의 학력을 가진 경우, 2, 3삼분기보다 1삼분기일 때 더 긍정적이었다.

남편의 성기능(IIEF)은 25점 만점에 평균 16.49±7.61점이었다. IIEF 점수는 임부의 출산력(t=2.36, *p*=.022)과 임신 기간(F=6.23, *p*=.002)에 따른 차이가 유의하였다. 즉 남편의 성기능은 임부가 출산경험이 없을 때보다 있을 때, 2삼분기보다 1삼분기일 때 더 높았다(Table 1).

### 임부와 남편의 임신 중 성에 대한 태도와 성기능 간의 상관관계

임부의 임신 중 성에 대한 태도와 성기능 간(r=.46, *p*<.001), 남편의 임신 중 성에 대한 태도와 성기능 간(r=.42, *p*<.001)에는 유의한 양의 상관관계가 있는 것으로 나타났다(Table 2).

### 임부와 남편의 성기능 영향요인

임신 중 성에 대한 태도가 성기능에 미치는 영향을 확인하기 위하여 성기능에 유의한 차이를 나타냈던 일반적 특성과 각자의 임신 중 성에 대한 태도를 독립변수로 투입하여 다변량 회귀분석을 실시하였다. 임부의 성기능 모델에는 교육 수준(1=전문대학교 졸업 이하)을 더미 변수로 처리하여 임신 중 성에 대한 태도(MSP)와 함께 투입하였다. 그 결과 Durbin-Watson 지수가 1.82로 오차 항들 간에 서로 독립적임을 확인하였고, tolerance (0.97)와 분산팽창지수(1.03)의 값을 통해 다중공선성이 없는 것으로 판단하였다. 임부의 성기능 모델은 통계적으로 유의하였고(F=24.68, *p*<.001) 전체 설명 변량은 20%였으며, 임신 중 성에 대한 태도가 긍정적인 임부의 성기능이 높게 나타났다(β=.44, *p*<.001).

남편의 성기능 모델을 검정하기 위하여 성기능에 유의한 차이를 나타낸 특성인 출산력(1=출산경험 있음)과 임신 기간(1=1삼분기, 2삼분기)을 더미 변수로 처리하여 임신 중 성에 대한 태도(PSP)와 함께 독립변수로 투입하였다. Durbin-Watson 지수가 1.83으로 오차 항들 간에 서로 독립적임을 확인하였고, tolerance (0.87–0.98)와 분산팽창지수(1.02–1.15) 값을 통해 다중공선성이 없는 것으로 판단하였다. 남편의 성기능 모델은 통계적으로 유의하였고(F=14.92, *p*<.001), 전체 설명 변량은 23%였으며, 임신 중 성에 대한 태도가 긍정적이고(β=.39, *p*<.001), 아내가 임신 3삼분기에 비해 1삼분기(β=.19, *p*=.004)인 남편의 성기능이 높게 나타났다(Table 3).

### 임부와 남편의 임신 전, 임신기간별 성행위의 변화

임부에게 임신 전 성행위에 대해 중복 응답하게 한 결과, 키스(93.5%), 질 삽입 성교(90.0%), 전희(82.3%), 구강성교(55.8%)의 순으로 빈도가 높았다. 1삼분기에는 키스(74.0%), 전희(55.4%), 질 삽입 성교(43.3%), 구강성교(17.3%) 순으로 높았다. 2삼분기에는 키스(46.3%), 질 삽입 성교(35.1%), 전희(34.2%), 구강성교(14.7%) 순으로 빈도가 높았다. 3삼분기에는 키스(27.7%), 전희(16.5%), 질 삽입 성교(15.2%), 구강성교(7.4%) 순으로 빈도가 높았다. 성행위를 하지 않는다는 응답은 3기(24.7%), 1기(20.3%), 2기(14.7%), 임신 전(0.9%) 순으로 높았다.

임신 중 성행위를 하지 않는 이유는 아기 손상의 두려움(40.7%), 질 출혈의 두려움(32.0%), 감염의 두려움(31.2%), 양막 파열의 두려움(29.0%) 순으로 높았다. 성행위를 한 후 경험한 부작용은 복부의 단단함(45.5%), 성교 통증(19.0%), 질 출혈(6.1%), 비뇨생식기계 감염(3.5%) 순으로 많았다. 임신 중 성생활에 대한 정보를 얻는 출처는 인터넷(74.5%)이 가장 많았으며 책(58.9%), 남편(20.8%), 의사(13.9%), 간호사(3.0%) 순이었다.

남편의 임신 전 성행위는 키스(91.3%), 질 삽입 성교(82.3%), 전희(81.4%), 구강성교(42.9%) 순이었고, 1삼분기에는 키스(68.4%), 전희(54.1%), 질 삽입 성교(45.0%), 구강성교(23.4%)의 순이었으며, 2삼분기에는 키스(43.7%), 질 삽입 성교(32.0%), 전희(27.7%)의 순이었고, 구강성교(10.8%)와 성적 상상(10.8%), 자위(10.8%)의 빈도가 같았다. 3삼분기에는 키스(28.6%), 질 삽입 성교(25.5%), 전희(16.0%), 자위(11.3%) 순이었다. 성행위를 하지 않는다는 응답은 3기(23.8%), 1기(18.6%), 2기(13.9%), 임신 전(1.3%) 순으로 높았다.

임부의 남편이 성행위를 하지 않는 이유로는 아기 손상의 두려움(41.1%), 감염의 두려움(33.8%), 양막 파열의 두려움(18.6%), 아내의 무성욕(13.4%) 순서로 높았다. 성행위 후 아내가 겪은 부작용에 대한 인식은 성교 통증(18.6%), 복부의 단단함(18.6%), 질 출혈(18.6%), 비뇨생식기계 감염(2.6%) 순으로 높았고, 임신 중 성생활에 대한 정보의 출처는 인터넷(74.0%), 아내(36.8%), 책(28.1%), 의사(20.3%), 간호사(6.1%) 순이었다(Table 4).

### 임부와 남편 간, 임신기간별 성기능부전 빈도의 비교

성기능 부전의 정도는 임부 74.9%, 남편 38.5%로 임부에게서 발생 빈도가 유의하게 높았다(χ^2^=62.21, *p*<.001). 임신 기간별로 살펴보면, 임부는 성기능 부전이 1삼분기에 90.4%로 가장 높았고 2삼분기에 72.7%, 3삼분기에 65.8%로 임신 기간이 지날수록 감소하였다(χ^2^=14.48, *p*=.001). 남편의 성기능 부전은 1삼분기에 27.4%로 가장 낮았다가 2삼분기에 52.3%로 가장 높았고, 3삼분기에 40.4%로 감소하였다(χ^2^=8.18, *p*=.017) (Table 5).

## Discussion

본 연구에서 임부의 성기능은 임신 기간에 따른 차이가 유의하지 않았으나 2, 3, 1삼분기 순서로 높았다. 이는 2삼분기[19]와 3삼분기[1]가 성기능의 위험요인으로 나타난 선행 연구들의 결과와 일치하지 않으나, Heidari 등[20]의 실험 연구에서 대조군의 성기능이 2, 3, 1삼분기 순서로 높게 나타난 결과와는 일치한다. 임신 기간과 성기능의 관련성에 대해서는 연구마다 일치하지 않는 결과를 보고하고 있으므로 큰 표본의 종단 연구를 통해 확인할 필요가 있다. 한편 임부의 성기능 평균 점수는 12점으로 성기능 부전의 절사점인 21점에 비해 매우 낮은 점수였고 그 결과 임부의 성기능 부전 비율이 75%로, 국내 기혼 여성의 57.5%[21]에 비해 높게 조사되었다. 본 연구의 임신 중 성에 대한 태도 이외에도 임부의 성기능 부전에 영향을 미치는 요인들을 다양한 영역에서 추가적으로 탐색할 필요가 있다.

임부와 남편의 임신 중 성에 대한 태도는 각자의 성기능과 유의한 양의 상관관계를 나타내었다. 이는 임신 중 성에 대한 태도와 성 만족 간에 유의한 양의 상관관계를 보고한 선행 연구[8]의 결과와 유사하다. 본 연구에서는 임신 중 성에 대한 태도가 임부와 남편의 성기능의 영향요인인 것으로 확인되었고, 남편의 경우에는 추가적으로 임신 기간도 영향을 미치는 것으로 나타났다. 선행 연구[8]에서도 임부와 남편의 성 태도는 각자의 성 만족의 영향요인으로 나타나 본 연구의 결과와 유사하였고, 상대방 효과를 조사하였을 때 남편의 성 태도는 임부의 성 만족에 영향을 주는 반면에 임부의 성 태도는 남편의 성 만족에 영향을 미치지 않음을 보고하였다. 한편 선행 연구에서 임신 중 성에 대한 임부와 파트너의 태도는 서로 강한 상관관계를 가진 것으로 나타났다[10]. 본 연구에서는 임신 중 성에 대한 태도와 성기능의 상대방 효과를 확인하려고 하였으나 2차에 걸쳐 자료 수집을 진행하는 동안 임부와 남편의 매칭에 혼란이 생겨 아쉽게도 확인할 수 없었다. 상대방 효과를 확인하는 후속 연구가 이루어지길 기대한다.

임신 중 성에 대한 태도는 성관계에 대한 어색함과 불안, 흥분되는 성생활에 대한 낮은 기대, 유산에 대한 두려움 등으로 이루어져 있다. 이를 긍정적인 방향으로 변화시키기 위해서는 생식기 해부학과 성 반응 주기 및 그에 대한 임신의 영향 등 성 생리학, 성행위에 대한 임신의 영향, 성교 기술, 임신 중 안전한 성 체위, 임신 중 성교 결과로 인한 인한 유산과 조기 파막의 위험 등에 대한 정보를 제공할 필요가 있다[20]. 임신 10–12주의 초임부와 초임부 커플 두 그룹을 대상으로 모형 활용 강의와 유인물 제공, 전화상담 중재를 90분씩 일주일 간격으로 2회 실시한 결과, 중재 4주 후와 2삼분기 말에 성기능에 유의한 효과가 있었다[20]. 본 연구에서 1삼분기는 임부와 남편 간 성 부조화가 가장 심한 시기이므로 임신 부부를 대상으로 한 성교육은 임신 초기부터 시행될 필요가 있다. 한편 이 시기는 산전 관리를 위해 병원을 자주 방문하는 시기는 아니므로 대면 교육 대신에 온라인이나 모바일로 제공되는 성교육 프로그램이 필요하다고 생각된다. 특히 남편의 성 태도는 임부의 성 만족에도 영향을 미치므로[8] 임부뿐만 아니라 남편에게도 함께 적용하는 것이 효율적일 것이다.

본 연구에서 임부들은 임신 전에 비해 임신 후 전반적으로 성행위의 감소를 보였으며, 임신 동안에는 기간이 지날수록 질 삽입 성교를 포함한 전반적인 성행위가 감소하였다. 질 삽입 성교를 보면, 1삼분기 43.3%, 2삼분기 35.1%, 3삼분기 15.2%로, 1삼분기에 가장 많이 수행하였다. 선행 연구[4]에서는 최근 4주 동안의 질 삽입 성교 횟수를 조사하였는데 임신 전 8.6회, 1–3삼분기에 각각 6.9회, 5.4회, 2.5회인 것으로 보고되었고, 또 다른 연구[22]에서도 임신이 경과할수록 감소를 보인 점은 본 연구와 일치하였다. 산과 간호사는 임신 인지 이후에도 성생활을 지속하는 임부의 성기능이 좋으며[11], 임신이 경과할수록 부부의 성관계가 소원해질 수 있다는 사실을 인식하고 산전관리 내용에 성교육과 성상담을 포함할 필요가 있다.

본 연구에서 1삼분기는 임부에게서 성기능 부전이 가장 많이 발생한 시기인 반면 남편의 경우에는 가장 적게 발생하는 시기로 나타나 부부 간 성 부조화의 위험이 1삼분기에 높을 것으로 예상된다. 본 연구에서 임신 중 성에 대한 태도는 성기능의 중요 요인으로 나타났는데, 1삼분기에 임신 중 성에 대한 태도가 아내는 가장 부정적인 반면에 남편은 가장 긍정적이기 때문에 나타난 결과로 보인다. 성적 만족은 결혼생활의 질에 기여하여 결혼의 안정성을 높이므로[23] 커플을 대상으로 1–3삼분기에 걸친 종단연구를 통해 임신 동안의 성 부조화 발생 현황을 정확하게 파악할 필요가 있다. 한편 임신 초기, 중기, 말기 간에 성 태도의 차이가 없고[24], 2삼분기와 3삼분기 간에 임신 중 성에 대한 태도가 유사하다는 보고가 있었으나[11], 본 연구에서는 1, 2삼분기에 비해 3삼분기의 태도가 유의하게 긍정적인 것으로 나타났다. 선행 연구들에서는 임신 중 성에 대한 태도를 조사하지 않았고[24], 2삼분기 임부의 표본이 적고 1삼분기 임부를 포함하지 않았기 때문에[11] 본 연구와 다른 결과를 나타낸 것으로 생각된다. 성관계를 금하라는 의사의 조언을 듣는 경우 임신 중 성에 대한 태도가 부정적이 되는데[10], 3삼분기는 유산과 조산에 대한 우려에서 벗어난 시기이며, 의사들도 이 시기에는 분만 촉진을 위해 성관계를 권장하기도 하므로[19] 긍정적인 태도를 나타낸 것으로 생각된다.

본 연구는 일 지역의 여성전문병원과 보건소를 이용하는 임신 부부를 편의 표출한 자료에 기반하므로 연구결과를 일반화하는데 제한이 있다. 그럼에도 불구하고 임부와 남편 모두를 대상으로 임신 중 성에 대한 태도가 성기능에 미치는 영향, 임신기간별 성행위의 변화, 성별과 임신기간에 따른 성기능부전 빈도의 차이를 밝힘으로써 임신 부부의 성중재 개발을 위한 기초 자료를 제공한 점은 본 연구의 의의라 할 수 있다. 다양한 영역에서 임신 부부의 성기능 영향요인에 대한 추가적인 탐색이 이루어진다면 임신 부부의 성기능 중재를 가이드 할 유용한 이론이 개발될 수 있으리라 기대한다.

부부 사이의 임신 중 성에 대한 태도와 성기능의 갭(gap)은 1삼분기에 가장 크게 나타났으므로 임신 부부를 위한 성 중재는 임신 초기 커플을 중심으로 이루어질 필요가 있다. 임신 부부의 임신 중 성에 대한 태도는 각자의 성기능의 영향요인으로 작용하고 있으므로 임신 중 성에 대한 태도를 긍정적인 방향으로 이끌 수 있는 중재 개발이 필요하다.

본 연구의 결과를 바탕으로 다음을 제언한다. 첫째, 임신 부부의 성기능을 이해하기 위해서는 호르몬이나 신체의 변화와 같은 생리적 영역, 부부 간 친밀도나 의사소통과 같은 심리사회적 영역 등 다양한 영역에서 요인을 탐색할 필요가 있다. 둘째, 임신 부부의 성기능의 상호적인 영향을 파악하기 위해서는 임신 중 성에 대한 태도와 성기능의 상대방에 대한 효과를 확인할 필요가 있다. 셋째, 임신 초기 부부를 대상으로 임신 중 성에 대한 태도를 긍정적으로 바꾸는 성교육 프로그램의 개발과 적용 연구를 제안한다.

## Figures and Tables

**Table 1.** Differences in sexual attitude and function by general characteristics (N=462)

Characteristic	Categories	n (%) or Mean±SD	MSP/PSP		FSFI/IIEF	
			Mean±SD	t/F (*p*)	Mean±SD	t/F (*p*)
Pregnant women (n=231)						
Age (year)	20–29	55 (23.8)	3.08±0.72	1.50 (.226)	13.50±6.07	1.47 (.224)
	30–39	165 (71.4)	2.86±0.87		11.34±6.93	
	≥40	11 (4.8)	2.78±0.91		14.13±7.66	
		32.19±4.09	2.91±0.84		12.01±6.79	
Education	≤College	95 (41.1)	2.99±1.23	2.29 (.023)	13.59±6.83	2.62 (.010)
	≥University	136 (58.9)	2.62±1.22		10.87±6.71	
Job	Yes	98 (42.4)	2.33±1.14	4.92 (<.001)	10.95±6.53	1.92 (.056)
	No	133 (57.6)	3.10±1.20		12.85±6.92	
Parity	0	195 (84.4)	2.80±1.20	0.41 (.679)	11.56±6.96	0.14 (.887)
	≥1	36 (15.6)	2.70±1.30		11.35±6.89	
Trimester	First ^a^	73 (31.6)	2.20±0.95	21.54 (<.001)	11.32±6.45	1.50 (.226)
	Second ^b^	44 (19.0)	2.45±1.23	a,b<c^†^	13.69±6.87	
	Third ^c^	114 (49.4)	3.26±1.20		11.85±7.01	
Husbands (n=231)						
Age (year)	20–29	20 (8.7)	3.68±0.74	0.76 (.468)	20.00±7.23	2.85 (.060)
	30–39	177 (76.6)	3.44±0.88		16.33±7.52	
	≥40	34 (14.7)	3.55±1.10		15.00±7.84	
		35.08±4.30	3.47±0.90		16.49±7.61	
Education	≤College	103 (44.6)	3.64±0.93	2.47 (.014)	17.34±6.86	1.37 (.171)
	≥University	126 (54.4)	3.34±0.86		15.97±8.09	
Job	Yes	224 (97.0)	3.45±0.91	1.13 (.260)	16.76±7.42	1.90 (.058)
	No	7 (3.0)	3.84±0.65		10.83±11.32	
Parity	0	195 (84.6)	3.37±0.88	0.74 (.466)	16.06±7.91	2.36 (.022)
	≥1	36 (15.6)	3.53±1.12		19.00±5.79	
Trimester	First ^a^	73 (31.6)	3.77±0.81	7.24 (.001)	18.73±5.53	6.23 (.002)
	Second ^b^	44 (19.0)	3.18±0.98	a>b,c^†^	13.84±8.98	a>b^†^
	Third ^c^	114 (49.4)	3.38±0.88		16.05±7.86	

FSFI: Female sexual function index; IIEF: international index of erectile function; MSP: Maternal sex during pregnancy scale; PSP: paternal sex during pregnancy scale.

**Table 2.** Correlations between the analyzed variables (N=462)

Couple	Variable	r (*p*)	
		MSP	PSP
Pregnant women (n=231)	FSFI	.46 (<.001)	
Husbands (n=231)	IIEF		.42 (<.001)

FSFI: Female sexual function Index; IIEF: international index of erectile function; MSP: maternal sex during pregnancy scale; PSP: paternal Sex during pregnancy scale.

† Scheffe test.

**Table 3.** Factors associated with sexual function (N=462)

Couple	Variable	B	SE	β	t	*p*
Pregnant women (n=231)	(Constant)	3.20	2.24		1.43	.155
	Education^†^	0.85	0.79	.07	1.07	.285
	MSP	0.58	0.09	.44	6.65	<.001
		R^2^=.21, adjusted R^2^=.20, F=24.68, *p*<.001				
		Durbin-Watson=1.82, tolerance=0.97, VIF=1.03				
Husbands (n=231)	(Constant)	4.56	1.92		2.38	.018
	Parity^†^	2.15	1.26	.11	1.71	.089
	First trimester^†^	2.91	1.14	.17	2.54	.012
	Second trimester^†^	–0.78	1.28	–.04	–0.61	.541
	PSP	3.19	0.54	.39	5.95	<.001
		R^2^=.25, adjusted R^2^=.23, F=14.92, *p*<.001				
		Durbin-Watson=1.83, tolerance=0.87–0.98, VIF=1.02–1.15				

MSP: Maternal Sex during Pregnancy Scale; PSP: paternal Sex during Pregnancy Scale; SE: standard error; VIF: variance inflation factor.

† Dummy variable references were education (≥university), parity (nullipara), and trimester (third).

**Table 4.** Changes in couples' sexual behavior before and during pregnancy

Variable	Categories	Pregnant women (n=231), n (%)				Husbands (n=231), n (%)			
		BP	First T	Second T	Third T	BP	First T	Second T	Third T
Sexual activities during pregnancy ^†^	Kissing	216 (93.5)	171 (74.0)	107 (46.3)	64 (27.7)	211 (91.3)	158 (68.4)	101 (43.7)	66 (28.6)
	Vaginal coitus	208 (90.0)	100 (43.3)	81 (35.1)	35 (15.2)	190 (82.3)	104 (45.0)	74 (32.0)	59 (25.5)
	Foreplay	190 (82.3)	128 (55.4)	79 (34.2)	38 (16.5)	188 (81.4)	125 (54.1)	64 (27.7)	37 (16.0)
	Cunnilingus	129 (55.8)	40 (17.3)	34 (14.7)	17 (7.4)	99 (42.9)	54 (23.4)	25 (10.8)	19 (8.2)
	Erotic fantasy	47 (20.3)	24 (10.4)	17 (7.4)	10 (4.3)	78 (33.8)	40 (17.3)	25 (10.8)	22 (9.5)
	Masturbation	24 (10.4)	6 (2.6)	8 (3.5)	6 (2.6)	91 (39.4)	48 (20.8)	25 (10.8)	26 (11.3)
	Anal sex	6 (2.6)	1 (0.4)	1 (0.4)	1 (0.4)	7 (3.0)	3 (1.3)	0 (0.0)	0 (0.0)
	No sexual life	2 (0.9)	47 (20.3)	34 (14.7)	57 (24.7)	3 (1.3)	43 (18.6)	32 (13.9)	55 (23.8)
		During pregnancy				During pregnancy			
Reasons for not having sex	Fear of injuring the baby	94 (40.7)				95 (41.1)			
	Fear of vaginal bleeding	74 (32.0)				1 (0.4)			
	Fear of infection	72 (31.2)				78 (33.8)			
	Fear of membrane rupture	67 (29.0)				43 (18.6)			
	One's decreased libido	51 (22.1)				14 (6.1)			
	Partner's decreased libido	11 (4.8)				31 (13.4)			
	Not knowing the feasibility of sex	11 (4.8)				15 (6.5)			
Maternal complications of sexual activities^†^	Abdominal hardness	105 (45.5)				32 (13.9)			
	Dyspareunia	44 (19.0)				75 (32.5)			
	Vaginal bleeding	14 (6.1)				29 (12.6)			
	Urogenital infection	8 (3.5)				6 (2.6)			
Information source^†^	Internet	172 (74.5)				171 (74.0)			
	Books	136 (58.9)				65 (28.1)			
	Spouse	48 (20.8)				85 (36.8)			
	Doctor	32 (13.9)				47 (20.3)			
	Nurse	7 (3.0)				14 (6.1)			

BP: Before pregnancy; T: trimester.

† Multiple response.

**Table 5.** Differences of couples' sexual dysfunction by trimester (N=462)

Couple	Sexual dysfunction		Trimester	Sexual dysfunction	
	n (%)	χ^2^(*p*)		n (%)	χ^2^(*p*)
Pregnant women (n=231)	173 (74.9)	62.21 (<.001)	First (n=73)	66 (90.4)	14.48 (.001)
			Second(n=44)	32 (72.7)	
			Third(n=114)	75 (65.8)	
Husbands (n=231)	89 (38.5)		First(n=73)	20 (27.4)	8.18 (.017)
			Second (n=44)	23 (52.3)	
			Third(n=114)	46 (40.3)	

## References

[1] Jamali S, Mosalanejad L (2013). Sexual dysfnction in Iranian pregnant women. Iran J Reprod Med.

[2] Aydin M, Cayonu N, Kadihasanoglu M, Irkilata L, Atilla MK, Kendirci M (2015). Comparison of sexual functions in pregnant and non-pregnant women. Urol J.

[3] Yoo O (2017). Factors that affect marital satisfaction of spouses in marriage for 5 years or less and 6 years or more. The Journal of Pastoral Care and Counseling. J Pastor Care Couns.

[4] Aslan G, Aslan D, Kizilyar A, Ispahi C, Esen A (2005). A prospective analysis of sexual functions during pregnancy. Int J Impot Res.

[5] Radoš SN, Vraneš HS, Šunjić M (2014). Limited role of body satisfaction and body image self-consciousness in sexual frequency and satisfaction in pregnant women. J Sex Res.

[6] Lemer JL, Blodgett Salafia EH, Benson KE (2013). The relationship between college women’s sexual attitudes and sexual activity: The mediating role of body image. Int J Sex Health.

[7] Sierra JC, Vallejo-Medina P, Santos-Iglesias P, Moyano N, Granados MR, del Mar Sánchez-Fuentes M (2014). [Sexual functioning in the elderly: Influence of age and psychosexual factors]. Rev Int Androl.

[8] Kim HE, Yeo JH (2017). Impact of sexual attitude and marital intimacy on sexual satisfaction in pregnant couples: an application of the actor-partner interdependence model. Korean J Women Health Nurs.

[9] Kaestle CE, Evans LM (2018). Implications of no recent sexual activity, casual sex, or exclusive sex for college women's sexual well-being depend on sexual attitudes. J Am Coll Health.

[10] Jawed-Wessel S, Herbenick D, Schick V, Fortenberry JD, Cattelona G, Reece M (2016). Development and validation of the maternal and partner sex during pregnancy scales. J Sex Marital Ther.

[11] Oh EJ, Kim MJ (2019). Factors affecting the sexual function of pregnant women. Korean J Women Health Nurs.

[12] Corbacioglu Esmer A, Akca A, Akbayir O, Goksedef BP, Bakir VL (2013). Female sexual function and associated factors during pregnancy. J Obstet Gynaecol Res.

[13] Pauleta JR, Pereira NM, Graça LM (2010). Sexuality during pregnancy. J Sex Med.

[14] Torkestani F, Hadavand SH, Khodashenase Z, Besharat S, Davati A, Karimi Z (2012). Frequency and perception of sexual activity during pregnancy in iranian couples. Int J Fertil Steril.

[15] Kim MJ, Kim MS, Kim YH, Lee YM, Oh EJ A validation study of the maternal and partner sex during pregnancy scale in Korean couples.

[16] Lee Y, Lim MC, Joo J, Park K, Lee S, Seo S (2014). Development and validation of the Korean version of the Female Sexual Function Index-6 (FSFI-6K). Yonsei Med J.

[17] Ahn TY, Lee DS, Kang WC, Hong JH, Kim YS (2001). Validation of an abridged Korean version of the International Index of Erectile Function (IIEF-5) as a diagnostic tool for erectile dysfunction. Korean J Urol.

[18] Jawed-Wessel S, Santo J, Irwin J (2019). Sexual activity and attitudes as predictors of sexual satisfaction during pregnancy: a multi-level model describing the sexuality of couples in the first 12 weeks. Arch Sex Behav.

[19] Abouzari-Gazafroodi K, Najafi F, Kazemnejad E, Rahnama P, Montazeri A (2015). Demographic and obstetric factors affecting women's sexual functioning during pregnancy. Reprod Health.

[20] Heidari M, Aminshokravi F, Zayeri F, Azin SA (2018). Effect of sexual education on sexual function of Iranian couples during pregnancy: a quasi experimental study. J Reprod Infertil.

[21] Kim HY, Lee ES (2010). Sexual dysfunction and related factors in married Korean women. J Korean Acad Psychiatr Ment Health Nurs.

[22] Gałązka I, Drosdzol-Cop A, Naworska B, Czajkowska M, Skrzypulec-Plinta V (2015). Changes in the sexual function during pregnancy. J Sex Med.

[23] Ozgoli G, Sheikhan Z, Zahiroddin A, Khodakarami N, Nasiri M (2019). The relationship of marital quality and sexual satisfaction with marital status in Iranian women: a Path model. Int J Appl Behav Sci.

[24] Lee YP, Kim SJ, Jeong GH (2000). Pregnant women's attitude and satisfaction for sexuality. J Korean Acad Nurs.

